# Duration of rifampin therapy is a key determinant of improved outcomes in early-onset acute prosthetic joint infection due to *Staphylococcus* treated with a debridement, antibiotics and implant retention (DAIR): a retrospective multicenter study in France

**DOI:** 10.7150/jbji.40333

**Published:** 2020-02-10

**Authors:** A. Becker, L. Kreitmann, C. Triffaut-Fillit, F. Valour, E. Mabrut, E. Forestier, O. Lesens, C. Cazorla, S. Descamps, B. Boyer, C. Chidiac, S. Lustig, E. Montbarbon, C. Batailler, T. Ferry

**Affiliations:** 1 Service des Maladies Infectieuses et Tropicales, Centre hospitalier universitaire (CHU) de la Croix Rousse, Hospices Civils de Lyon (HCL), Lyon (France); 2 Centre de Référence des Infections Ostéo-Articulaires Complexes (CRIOAc) de Lyon (France); 3 Service de Réanimation Médicale, Hospices Civils de Lyon, Hôpital Edouard Herriot, Lyon, France; 4 Université Claude Bernard Lyon 1, Lyon, France; 5 Inserm U1111, Centre international de recherche en Infectiologie (CIRI), Université Claude-Bernard Lyon 1, Lyon, France; 6 Service des Maladies Infectieuses et Tropicales, Centre hospitalier Métropole Savoie, Chambéry (France); 7 Service des Maladies Infectieuses et Tropicales, Centre hospitalier universitaire (CHU) Gabriel Montpied, Clermont-Ferrand (France); 8 Service des Maladies Infectieuses et Tropicales, Centre hospitalier universitaire (CHU) de Saint-Etienne (France); 9 Service de Chirurgie Orthopédique, Centre hospitalier universitaire (CHU) Gabriel Montpied, Clermont-Ferrand (France); 10 Service de Chirurgie Orthopédique, Centre hospitalier universitaire (CHU) de Saint-Etienne (France); 11 Service de Chirurgie Orthopédique, Centre hospitalier universitaire (CHU) de la Croix Rousse, Hospices Civils de Lyon (HCL), Lyon (France); 12 Service de Chirurgie Orthopédique, Centre hospitalier Métropole Savoie, Chambéry (France)

**Keywords:** debridement, antibiotics and implant retention (DAIR), prosthetic joint infection, rifampin, *Staphylococcus aureus*, coagulase negative staphylococci

## Abstract

**Introduction**: In patients undergoing a « debridement, antibiotics, and implant retention » (DAIR) procedure for acute staphylococcal prosthetic joint infection (PJI), post-operative treatment with rifampin has been associated with a higher probability of success.(1,2) However, it is not known whether it is the total dose, delay of introduction or length of therapy with rifampin that is most strongly associated with the observed improved outcomes.

**Methods**: A multicentric, retrospective cohort study of patients with acute staphylococcal hip and knee PJI treated with DAIR between January 2011 and December 2016. Failure of the DAIR procedure was defined as persistent infection, need for another surgery or death. We fitted logistic and Cox regression multivariate models to identify predictors of DAIR failure. We compared Kaplan-Meier estimates of failure probability in different levels of the 3 variables of interest - total dose, delay of introduction or length of therapy with rifampin - with the log-rank test.

**Results**: 79 patients included (median age 71 years [63.5-81]; 55 men [70%]), including 54 (68%) DAIR successes and 25 (32%) DAIR failures. Patients observed for a median of 435 days [IQR 107.5-834]. Median ASA score significantly lower in DAIR successes than in DAIR failures (2 vs. 3, respectively *p* = 0.011). Bacterial cultures revealed 65 (82.3%) *S. aureus* and 16 (20.3%) coagulase negative staphylococci, with 2 patients being infected simultaneously with *S. aureus* and CNS. Among *S. aureus* isolates, 7 (10.8%) resistant to methicillin; 2 (3.1 %) resistant to rifampin. Median duration of antimicrobial therapy was 85 days [IQR 28.5-97.8]. Fifty-eight patients (73.4%) received rifampin at a median dose of 14.6 mg/kg/day |IQR 13-16.7], started at a median delay of 8.5 days [IQR, 4-7.5] after debridement surgery. Twenty-one patients (26.6%) developed a drug-related adverse event, leading to rifampin interruption in 6 of them (7.6% of total cohort). Determinants of DAIR failure were rifampin use (HR 0.17, IC [0.06, 0.45], p-value <0.001), association of rifampin with a fluoroquinolone (HR 0.19, IC [0.07, 0.53], p-value = 0.002) and duration of rifampin therapy (HR 0.97, IC [0.95, 1], p-value = 0.022). We did not observe a significant difference between DAIR successes and failures in rifampin use, dose and delay of introduction. In a multivariate Cox model, only duration of rifampin therapy was significantly associated with DAIR failure. Kaplan Meier estimate of DAIR failure probability was significantly higher in patients receiving less than 14 days of rifampin in comparison with those receiving more than 14 days of rifampin (*p* = 0.0017).

**Conclusion**: Duration of rifampin therapy is a key determinant of improved outcomes in early-onset acute prosthetic joint infection due to *Staphylococcus* treated with DAIR.

## Introduction

Successful joint replacement has been shown to provide pain relief, restore mobility and improve quality of life in millions of patients. It has been projected that respectively 572,000 and 3.48 million hip and knee arthroplasty procedures will be performed in the United States in 2030 3. While only a minority of joint prostheses ever become infected, prosthetic joint infections (PJI) are responsible for a huge burden for patients and significant costs for the healthcare system 3.

Early-onset PJI is defined as a PJI occurring within 3 months following index arthroplasty 4, while the term « acute PJI » is used for patient with a short duration of symptoms (< 30 days)^5^. *Staphylococcus aureus* and coagulase-negative staphylococci (CNS) are the most frequent bacteria encountered in this setting 6,7, accounting respectively for 38% and 27% of all hip and knee early-onset PJI 3.

Successful management of PJI requires surgical intervention and medical therapy in all cases, but the choice of the best strategy is challenging. A « debridement, antibiotics, and implant retention » (DAIR) procedure is indicated in patients with a short duration of symptoms (< 30 days), a stable implant and no sinus tract 4. This strategy is attractive because it can preserve implant function and bone mass, while it reduces the need for further surgical intervention 8.

According to the Infectious Diseases Society of America (IDSA) guidelines, the optimal management of acute staphylococcal PJI treated with the DAIR procedure should be based on a prolonged antibiotic combination therapy with rifampin 4. This strategy is supported by the results of one small randomized controlled trial 2 and several observational studies 1,5,9. However, the use of rifampin in this clinical setting can be challenging, as some patients cannot receive it due to drug-drug interaction, and many others have to stop it due to drug-related adverse reactions. Furthermore, while the clinical benefit of rifampin in this context has been extensively documented, it is still not known whether it is the total dose, duration of therapy and/or delay of introduction of rifampin that have the most significant impact on the DAIR procedure probability of success. Thus, the aim of the study was to investigate which of these three factors - total dose, duration of therapy and delay of introduction - was most strongly associated with the improved outcomes observed with rifampin use in patients treated with a DAIR procedure for acute staphylococcal PJI.

## Methods

### Study setting and patient inclusion criteria

We performed a retrospective cohort study in four hospitals of the Auvergne-Rhône-Alpes region in France, including three university-affiliated hospitals with two reference centers for the management of complex bone and joint infections, and one general hospital. Patients with a diagnosis of early-onset acute PJI due to *Staphylococcus* sp. and treated with DAIR between January 2011 and December 2016 were eligible for the study. Early-onset acute PJI was defined as occurring with one month following index arthroplasty. The diagnosis of PJI was made according to international guidelines 4. Specifically, PJI was suspected in patients with post-operative persistent wound drainage, sinus tract, local signs of joint infection, acute onset of painful prosthesis, or general symptoms of infection. *S. aureus* PJI was defined as the isolation of ≥ 1 strain of *S. aureus* from a reliable sample taken from the prosthetic site, and coagulase negative staphylococcal PJI was defined as ≥ 2 reliable samples positive for the same strain of any coagulase negative *Staphylococcus* species. PJI due to a *Staphylococcus* strain and another non-Staphylococcus bacteria or with fungi were excluded. Cases were identified from previously registered PJI databases or from the general archives of each hospital. Patients were observed from the time of debridement surgery to most recent contact.

### Medical and surgical procedures

As patients with unstable prostheses are not considered good candidates to a DAIR procedure, these were not recruited in our study 6. More generally, the decision to undergo DAIR and the choice of antimicrobial therapy were made by the attending medical team, based upon current national 10 and international practice guidelines 4. A combination of antimicrobial agents administered intravenously was begun intraoperatively immediately after samples were taken. It consisted of a broad-spectrum β-lactam agent and a second antimicrobial agent with activity against methicillin-resistant staphylococci. This treatment was continued until microbiological results of the preoperative sample culture were available and was then modified on the basis of culture results.

### Data collection

We reviewed medical charts of patients fulfilling the inclusion criteria and recorded the following variables: baseline patient demographics and comorbidities; characteristics of initial arthroplasties; microbiological samples collected upon debridement surgery; characteristics of the debridement surgery; post-operative antibiotic therapy. We recorded whether the initial arthroplasty was a primary or a revision implantation (the latter case being due to non-septic loosening of the initial prosthesis). Time to infection was defined as the time between initial arthroplasty to clinical onset of infection. Use, duration, doses, delay of introduction of rifampin and its combination with fluoroquinolones were more specifically recorded. Failure of the DAIR procedure was defined as: 1) prosthesis removal for any cause during the follow-up period (including prosthesis superinfection with another bacteria/fungi or recurrence of staphylococcal infection); or 2) the need for an additional debridement before completion of antibiotic therapy (including in case of evacuation of a post-operative hematoma within 48 hours following debridement); or 3) the need for additional antibiotic therapy after completion of the first course, including the requirement of a prolonged suppressive antibiotic therapy in patients unable to undergo revision surgery; or 4) the existence of clinical or microbiological signs of infection within 2 years after debridement; or 5) death with signs of infection or before completion of antibiotic therapy. Drug-related adverse event was defined as any undesirable experience occurring under drug-therapy. In patients with a failure of the DAIR procedure, we recorded time between debridement surgery and failure; in patient with success of the DAIR procedure, we recorded time between debridement surgery and most recent contact and the follow-up data was then right-censored.

### Statistical analysis

We compared baseline characteristics, surgical procedures, microbiological data and antibiotic therapy according to outcome. Results are expressed as numbers (percentages) for categorical variables and as median (interquartile range [IQR]) for continuous variables. For the percentage calculation, the number of missing values was excluded from the denominator. Categorical variables were compared using Pearson χ^2^ test or Fisher exact test, as indicated, and continuous variables were compared using the Wilcoxon signed-rank test. We fitted univariate Cox proportional hazards models to identify independent predictors of treatment failure. To this end, we did not include in our analysis DAIR failures occurring while the patient was still under rifampin (to limit the risk of bias where the proportion of patients with a short duration of rifampin therapy is inflated among patients presenting with a DAIR failure). Variables of medical interest that had a significant effect on outcome with a p-value < 0.20 in univariate analysis were retained to fit a multivariate Cox regression model. In the multivariate model, a p-value < 0.05 was deemed statistically significant. We computed Kaplan-Meier estimates of failure probability and compared them in different levels of duration of rifampin therapy using the log-rank test. Statistical analysis was carried out using the SPSS and R softwares. No correction was made for multiple comparisons.

### Ethics

The study was conducted in accordance with the principles of the Declaration of Helsinki and local laws and guidelines for Good Clinical Practice. Written information was given to all patients and their consent obtained. The study was approved by the institutional review boards of all study hospitals.

## Results

As indicated in Table 1, 79 patients were included in our cohort between January 2011 and December 2016, including 54 DAIR successes and 25 DAIR failures. Patients were observed for a median of 435 days (IQR 107.5, 834). There were 55 men (70%) and median age was 71 [63.5, 81] years. Body mass index (BMI) was significantly lower in patients with a DAIR success than in those with a DAIR failure (26.5 vs. 29.1 kg/m2, respectively, p = 0.016). 17 patients (21.5%) had diabetes and 14 (17.7%) were active smokers. Median Charlson comorbidity index (CSI) was 4 [3, 5.5]. Median ASA score was significantly lower in DAIR successes than in DAIR failures (2 vs. 3, respectively p = 0.011). 59 patients (74.7%) had a hip PJI, while the remaining 21 (26.6%) had a knee PJI. Overall, infections followed a primary implantation in 67 cases (84.8%).

Bacterial cultures were positive with *S. aureus* in 65 cases (82.3 %) and with coagulase negative staphylococci (CNS) in 16 cases (20.3 %), with 2 patients being infected simultaneously with *S. aureus* and CNS. Among *S. aureus* isolates, 7 (10.8%) were resistant to methicillin; 2 (3.1 %) and 10 (15.4%) were resistant to rifampin and fluoroquinolones, respectively. We observed significantly more CNS isolates (and a trend towards less *S. aureus* isolates) in patients with a DAIR success than in those with a DAIR failure (15 vs. 1, p = 0.032).

Total duration of antimicrobial therapy was 85 days (IQR 28.5, 97.8), and it was significantly longer in DAIR successes than in DAIR failures (91.5 vs. 15 days, p < 0.001). Fifty-eight patients (73.4%) received rifampin at a median dose of 14.6 mg/kg/day (IQR 13, 16.7), and it was started a median 8.5 days (IQR, 4-7.5) after debridement surgery. We did not observe a significant difference between DAIR successes and failures in rifampin use, dose and delay of introduction. Association with a quinolones was more frequent in DAIR successes than in DAIR failures (57.4% vs. 20% respectively, p=0.004). Among the 58 patients treated with rifampin, 13 (22.4%) received the drug for less than 14 days. Twenty-one patients (26.6%) developed a drug-related adverse event, leading to rifampin interruption in 6 of them (7.6% of total cohort).

During debridement surgery, 77 patients (97.5%) underwent arthrotomy (the 2 remaining undergoing arthroscopy) and 38 of them (56.7%) had a removal of polyethylene components, with no difference between groups.

A total of 25 DAIR failures were recorded, with a median 16 days (IQR 14, 82) between debridement and failure. Among DAIR failures, we identified 12 cases of relapse (identification of the same bacteria as initial infection), while other 13 failures where due to infection with another bacterial strain.

**Figure 1:**
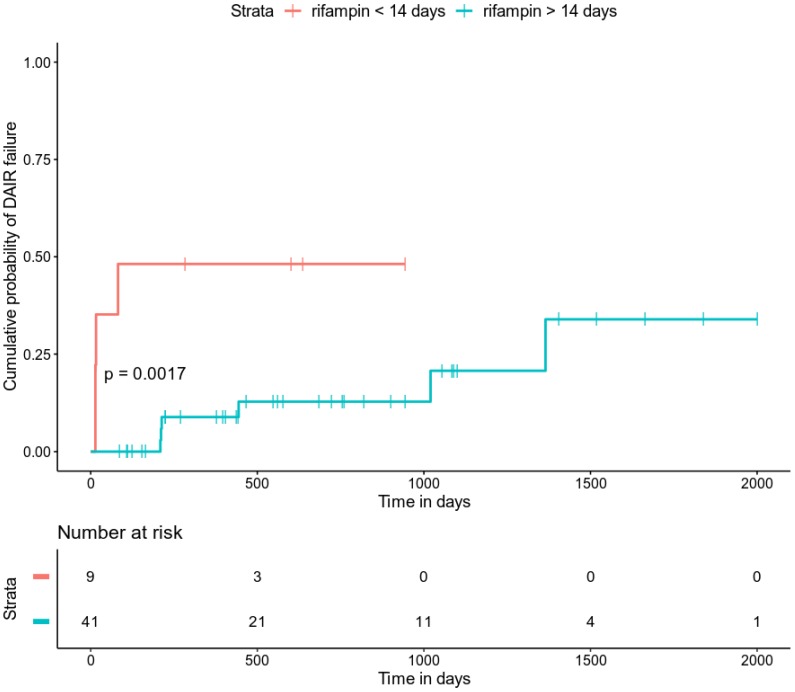
Kaplan Meier estimates of treatment failure probability in patients receiving less and more than 14 days of rifampin.

Factors associated with DAIR failure in univariate Cox regression analysis are presented in Table 2. Determinants of treatment failure were diabetes mellitus (HR 2.17, IC [0.82, 5.72], p-value = 0.118), active smoking (HR 2.26, IC [0.85, 6.04], p-value = 0.103), Charlson comorbidity index (HR 1.10, IC [0.97, 1.26], p-value = 0.130), ASA score (HR 2.07, IC [1.07, 4.03], p-value = 0.031), *Staphylococcus aureus* (HR 4.03, IC [0.54, 30.27], p-value 0.175), MRSA (HR 2.56, IC [0.73, 8.94], p-value = 0.140), total duration of antibiotic therapy (HR 0.98, IC [0.97, 0.99], p-value = 0.002), rifampin use (HR 0.17, IC [0.06, 0.45], p-value <0.001), association of rifampin with a fluoroquinolone (HR 0.19, IC [0.07, 0.53], p-value = 0.002) and duration of rifampin therapy (HR 0.97, IC [0.95, 1], p-value = 0.022). When these factors where included in a multivariate Cox model, only duration of rifampin therapy was significantly associated with DAIR failure (with a negative hazard-ratio indicating a higher probability of DAIR success with longer duration of rifampin therapy. As shown in Figure 1, Kaplan Meier estimate of treatment failure probability was significantly higher in patients receiving less than 14 days of rifampin in comparison with those receiving more than 14 days of rifampin (p = 0.0017).

## Discussion

This is a retrospective observational study in 79 patients with acute postoperative staphylococcal prosthetic joint infection (PJI). The aim of the study was to evaluate the impact of rifampin on the outcome of patients treated with debridement, antibiotics and retention (DAIR) according to current guidelines, more specifically to investigate whether it is the total dose, duration of therapy and/or delay of introduction of rifampin that have the most significant impact on the DAIR procedure probability of success. We found that duration of rifampin therapy was the variable that most significantly associates with a favorable outcome.

We found 68% success rate of the DAIR procedure in patients with acute early-onset staphylococcal PJI. This is in line with reported success rates of the DAIR procedure in this clinical setting, which have been found between 31% and 82% among infections with different pathogens 3,6,7, with an average rate of 52% for hip and knee arthroplasty in a systematic review 11 and 55% in a large retrospective multicenter study including patients with *S. aureus* PJI 9.

Second, we observed that factors associated with DAIR failure (both in a negative and a positive direction) were coherent with those reported in numerous studies by other groups, including active smoking, diabetes mellitus, ASA score, rifampin combination therapy with a fluoroquinolone 2,5,12. However, in our study, only duration of rifampin was significantly and positively associated with DAIR success in multivariate analysis.

Scheper et al. 13 reported that short-term postoperative treatment with rifampin resulted in high cure rates in patients with staphylococcal hip PJI, suggesting that prolonged treatment with rifampin might not be needed. Our study is in contradiction with these results, but several methodological limitations in the study by Scheper can be discussed: 1/ some patients with chronic biofilm infection were treated with DAIR; 2/ oral treatment was started after 2 weeks with flucloxacillin, with a risk of attaining low drug levels; and 3/ rifampin was started immediately after debridement, resulting in one failure with a rifampin-resistant *S. aureus*.

Our study has several limitations, most importantly its non-randomized and retrospective design, and the fact that comparisons were made on small samples. We cannot rule out a bias of reverse causality when assessing the association between a short duration of rifampin therapy and DAIR failure. Indeed, it could be objected that patients with a DAIR failure have a higher risk of stopping rifampin because of treatment failure (and not the other way around), thus leading consequently to a shorter duration of treatment with rifampin. However, we excluded from our analysis patients who failed during rifampin treatment to account for this risk of bias.

Furthermore, a retrospective data collection exposes to a risk of missing data. Specifically, we did not record information about companion drugs other than fluroquinolones, specific causes of drug-related adverse events and causes of rifampin discontinuation not related to an adverse event. We observed that 25% of patients in our study could not receive rifampin, and half of patients with a DAIR failure received less than 30 days of rifampin. This can appear contradictory to current guidelines 4,10,14. Furthermore, all cases of rifampin discontinuation were not formally explained in medical records. But this reflects "real life" data, and could be explained by other drawbacks associated with rifampin use, including its many drug-drug interactions.

## Conclusion

Our study suggests that duration of rifampin therapy is a key determinant of improved outcomes in early-onset acute prosthetic joint infection due to *Staphylococcus* treated with DAIR, whereas timing of initiation and dosing of rifampin did not significantly affect the outcome. These data show that additional prospective studies are warranted to elucidate the optimal duration of rifampin as part of the antimicrobial therapy in patients with a staphylococcal PJI. Furthermore, as 25% of patients could not receive rifampin, new drugs with anti-biofilm activity are required.

## Figures and Tables

**Table 1:** Baseline characteristics of patients included, microbiology data, therapeutic characteristics and follow-up

	DAIR success (n = 54)	DAIR failure (n = 25)	p-value
Sexe (male)	37 (68.5%)	18 (72%)	0.96
Age (years)	71.5 [64, 80.8]	71 [60, 81]	0.962
Body mass index (kg/m²)	26.5 [23.4, 28.7]	29.6 [26.8, 31.7]	0.016
**Comorbidities**			
Diabetes mellitus	9 (16.7%)	8 (32%)	0.212
Autoimmune disease	3 (5.6%)	0 (0%)	0.57
Active cancer	7 (13%)	1 (4%)	0.408
Active smoking	7 (13%)	7 (28%)	0.19
Charlson comorbidity index	4 [3, 5]	5 [2, 8]	0.598
ASA score	2 [2, 2]	3 [2, 3]	0.011
**Initial arthroplasty**			
Hip	40 (74.1%)	19 (76%)	1
Knee	15 (27.8%)	6 (24%)	0.936
Primary implantation	46 (85.2%)	21 (84%)	1
**Microbiology**			
*Staphylococcus aureus*	41 (75.9%)	24 (96%)	0.063
MSSA	38 (92.7%)	21 (87.5%)	0.8
MRSA	4 (9.8%)	3 (12.5%)	1
Rifampin-resistant *S. aureus*	2 (4.9%)	0 (0%)	0.723
Fluoroquinolone-resistant *S. aureus*	5 (12.2%)	5 (20.8%)	0.565
Coagulase negative staphylococci	15 (27.8%)	1 (4%)	0.032
MS-CNS	7 (53.8%)	0 (0%)	1
MR-CNS	6 (46.2%)	1 (100%)	1
Rifampin-resistant CNS	13 (100%)	1 (100%)	1
Fluoroquinolone-resistant CNS	5 (38.5%)	1 (100%)	0.881
**Antibiotic therapy**			
Total duration (days)	91.5 [72, 106]	15 [11, 44.3]	<0.001
Rifampin use	41 (75.9%)	17 (68%)	0.64
Rifampin + fluoroquinolone	31 (57.4%)	5 (20%)	0.004
Daily rifampin dose (mg/kg/day)	15.3 [13, 16.8]	13.9 [13, 15.4]	0.416
Delay between debridement and rifampin introduction (days)	10 [6.5, 21.5]	3 [1, 10]	0.008
Duration of rifampin (days)	75 [27.8, 91.2]	14.5 [10.2, 41]	0.001
Drug-related adverse event	18 (33.3%)	3 (12%)	0.085
Drug-related adverse event leading to rifampin interruption	6 (11.1%)	0 (0%)	0.202
**Debridement surgery**			
Removal of polyethylene components	28 (56%)	10 (58.8%)	1
Arthrotomy	52 (96.3%)	25 (100%)	0.838
**Time frame**			
Time to infection (days)	20.5 [16, 25.8]	21 [16, 27]	0.358
Time to outcome (days)	440.5 [153, 860.8]	16 [14, 82]	<0.001
Total follow-up (days)	440.5 [153, 860.8]	187 [25.5, 617]	0.089

ASA: American Society of Anesthesiologists (a clinical scoring system widely used to assess pre-operative clinical status); MSSA: methicillin-susceptible *Staphylococcus aureus;* MRSA: methicillin-resistant *Staphylococcus aureus*; MS-CNS: methicillin- susceptible coagulase negative staphylococci; MR-CNS: methicillin-resistant coagulase negative staphylococci

**Table 2:** Hazard-ratios of treatment failure in univariate Cox regression analysis

	HR	CI_95%_	p-value
Sex (male)	1.02	[0.98, 1.05]	0.326
Age (years)	0.84	[0.33, 2.16]	0.723
Body mass index (kg/m²)	1.00	[0.98, 1.02]	0.869
Diabetes mellitus	2.17	[0.82, 5.72]	0.118
Autoimmune disease	Inf	[0, Inf]	0.997
Active cancer	0.88	[0.62, 1.23]	0.441
Active smoking	2.26	[0.85, 6.04]	0.103
Charlson comorbidity index	1.10	[0.97, 1.26]	0.130
ASA score	2.07	[1.07, 4.03]	0.031
Hip	1.08	[0.39, 3.02]	0.885
Knee	0.88	[0.31, 2.46]	0.807
Primary implantation	1.25	[0.29, 5.41]	0.767
*Staphylococcus aureus*	4.03	[0.54, 30.27]	0.175
MRSA	2.56	[0.73, 8.94]	0.140
Total duration of antibiotic therapy (days)	0.98	[0.97, 0.99]	0.002
Rifampin use	0.17	[0.06, 0.45]	<0.001
Rifampin + fluoroquinolone	0.19	[0.07, 0.53]	0.002
Daily rifampin dose (mg/kg/day)	1.00	[0.88, 1.13]	0.958
Delay between debridement and rifampin introduction (days)	0.99	[0.95, 1.03]	0.488
Duration of rifampin (days)	0.97	[0.95, 1]	0.022
Drug-related adverse event	0.52	[0.15, 1.79]	0.299
Drug-related adverse event leading to rifampin interruption	Inf	[0, Inf]	0.998
Removal of polyethylene components	1.19	[0.36, 3.92]	0.778
Arthrotomy	Inf	[0, Inf]	0.998
Time to infection (days)	1.02	[0.94, 1.09]	0.674

HR: hazard-ratio; CI_95%_: 95% confidence interval; ASA: American Society of Anesthesiologists (a clinical scoring system widely used to assess pre-operative clinical status); MRSA: methicillin-resistant *Staphylococcus aureus*.

**Table 3:** Hazard-ratios of treatment failure in multivariate Cox regression analysis

	HR	CI95%	p-value
Diabetes mellitus	0.8	[0.08, 8.38]	0.855
Active smoking	2.69	[0.25, 28.7]	0.412
Charlson comorbidity index	1.26	[0.87, 1.84]	0.225
ASA score	2.67	[0.55, 13.0]	0.225
*Staphylococcus aureus*	2.7	[0.17, 42.1]	0.479
MRSA	2.29	[0.12, 42.1]	0.577
Total duration of antibiotic therapy (days)	1	[0.99, 1.01]	0.786
Rifampin use	Inf	[0.00, Inf ]	0.998
Rifampin + fluoroquinolone	0.28	[0.02, 3.83]	0.338
Duration of rifampin (days)	0.95	[0.92, 0.99]	0.022

HR: hazard-ratio; CI_95%_: 95% confidence interval; ASA: American Society of Anesthesiologists (a clinical scoring system widely used to assess pre-operative clinical status); MRSA: methicillin-resistant *Staphylococcus aureus*.
